# Digital dissection – using contrast-enhanced computed tomography scanning to elucidate hard-and soft-tissue anatomy in the Common Buzzard *Buteo buteo*

**DOI:** 10.1111/joa.12153

**Published:** 2013-12-18

**Authors:** Stephan Lautenschlager, Jen A Bright, Emily J Rayfield

**Affiliations:** School of Earth Sciences, University of BristolBristol, UK

**Keywords:** avian anatomy, interactive model, iodine staining, three-dimensional visualisation

## Abstract

Gross dissection has a long history as a tool for the study of human or animal soft-and hard-tissue anatomy. However, apart from being a time-consuming and invasive method, dissection is often unsuitable for very small specimens and often cannot capture spatial relationships of the individual soft-tissue structures. The handful of comprehensive studies on avian anatomy using traditional dissection techniques focus nearly exclusively on domestic birds, whereas raptorial birds, and in particular their cranial soft tissues, are essentially absent from the literature. Here, we digitally dissect, identify, and document the soft-tissue anatomy of the Common Buzzard (*Buteo buteo*) in detail, using the new approach of contrast-enhanced computed tomography using Lugol's iodine. The architecture of different muscle systems (adductor, depressor, ocular, hyoid, neck musculature), neurovascular, and other soft-tissue structures is three-dimensionally visualised and described in unprecedented detail. The three-dimensional model is further presented as an interactive PDF to facilitate the dissemination and accessibility of anatomical data. Due to the digital nature of the data derived from the computed tomography scanning and segmentation processes, these methods hold the potential for further computational analyses beyond descriptive and illustrative proposes.

## Introduction

**Figure 1:**
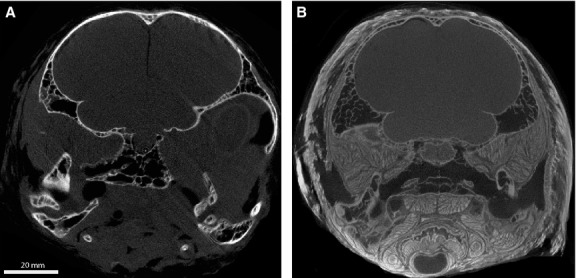
Coronal CT images showing a specimen of *Buteo buteo* (A) without and (B) with iodine staining.

**Figure 2:**
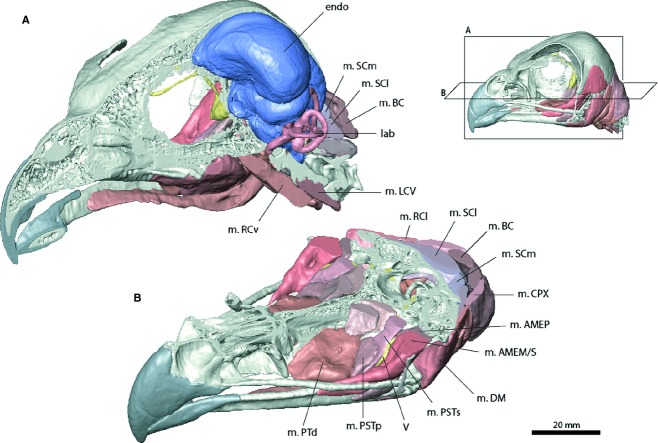
Visualised soft tissues of *Buteo buteo* in (A) sagittal and (B) horizontal cross-sections, including the jaw adductor musculature, various cervical muscles and the endocranial anatomy (brain and endosseous labyrinth based on casts of the cranial cavity).

Gross dissection is an established method for gaining detailed information about human or animal physiology, with a history stretching back many hundreds of years. Since then, different techniques such as histology or light microscopy have supplemented and refined this approach. Nevertheless, gross dissection has the disadvantage of being a time-intensive and destructive method. Once a specimen has been dissected, it is lost and cannot be re-examined to confirm observations. Recent advances in X-ray computed tomography (CT) scanning technologies and their increasing availability have led to a surge of alternative non-destructive imaging techniques in medical, biological and life sciences (Mizutani & Suzuki, 2012; Faulwetter et al. 2013). These techniques vastly enhance our capability to identify, visualise and quantify complex anatomical structures. However, due to low intrinsic X-ray absorption, CT and microCT scanning rarely provide sufficient resolution of unmineralised soft tissues (Fig. 1). Furthermore, similar attenuation between different tissue types impedes the automatic or manual differentiation between individual organs and tissues. Very recently, experimental studies have produced promising results using contrast-enhancing agents (Metscher, 2009a,b) to increase differential attenuation. In particular, iodine staining (sometimes referred to as Lugol's iodine or Lugol's solution) has been shown to represent a fast and inexpensive method of imparting high differential contrast with histological resolution (Jeffery et al. 2011). This method has been used effectively to investigate the comparative morphology of small vertebrate muscles, for which traditional dissection techniques are difficult (Cox & Jeffery, 2011; Baverstock et al. 2013) or to visualise the adductor musculature in some crocodilians (Tsai & Holliday, 2011; Holliday et al. 2013). The other living archosaur lineage, birds, has not been studied making use of this imaging technique. The technique readily lends itself to the detailed study of bird anatomy, due to the small size of many birds, because gross dissection of such specimens is often challenging, time-consuming, and requires considerable skill and experience. Furthermore, in-depth anatomical descriptions and documentations of avian anatomy at this level of detail and resolution are rarely seen, if at all. Classical works providing accurate descriptions and/or illustrations of the cranial hard-and soft-tissue anatomy in birds based on gross dissection have largely focused on domestic birds (Shufeldt, 1890; Ghetie et al. 1976; Baumel et al. 1993). Detailed studies of the cranial anatomy, and in particular the myology, of raptorial birds, however, are rare or only schematic (Hull, 1991; Sustaita, 2008; Onuk & Kabak, 2012). Given that the cranial musculature in birds is highly variable and that raptorial birds have a modified and specialised myological architecture (George & Berger, 1966; Bühler, 1981), contrast-enhanced CT scanning using iodine staining offers a powerful tool to visualise, document and describe their anatomy. To demonstrate this, we present the results of a ‘digital dissection’ of a Common Buzzard (*Buteo buteo*). The digital visualisation of contrast-enhanced soft tissues offers a unique opportunity to describe and illustrate a variety of soft-tissue structures in detail, and in an osteological context. Using this approach, the cranial musculature (jaw adductor and depressor, hyoid, and neck musculature), ligaments, endocranial and neurovascular structures, and keratinous tissues are digitally reconstructed and described here (Fig. 2). Furthermore, we assess the presence and the ability of osteological correlates to identify muscle adductor attachment and insertion sites, which has implications for the ability to reconstruct cranial soft tissues in extinct taxa.

## Material and methods

The freshly frozen head of an adult male Common Buzzard (*Buteo buteo*) was obtained from a local veterinary practice. The specimen was thoroughly defrosted before being submerged in a 10% solution of I_2_KI in 4% paraformaldehyde in phosphate-buffered saline, and stored in a refrigerator. After 7 days, the specimen was removed from the iodine solution and re-frozen for transport. Prior to CT scanning, the specimen was defrosted once more. This repeated freezing and thawing may have caused some shrinkage of the muscle tissues; however, as all the masticatory and cervical muscles that we have reconstructed are anchored to bones, muscle fibre lengths should not be affected. CT scanning of the specimen in air was performed on an X-Tek HMX 160 μCT system at the University of Hull, UK (X-Tek Systems Ltd., UK; resolution = 0.0581 mm, 95 kV, 60 μA). A scan of an unstained specimen in air that was used in a different study was also obtained from the same scanner, to allow comparisons between stained and unstained tissues (resolution = 0.0597 mm, 81 kV, 40 μA; Fig. 1).

The CT data files were imported into avizo (Version 7.0, Visualization Science Group) to identify and segment the anatomical structures of interest (e. g. bone, musculature and neurovascular structures). The segmentation was performed manually based on attenuation differences between bone and soft tissues in the iodine stained dataset. Three-dimensional (3D) surface models and volumes were created to visualise the segmented hard and soft tissues. Additionally, surface models of the individual structures were downsampled to a degree that allowed for small files sizes but preserved all details, and were exported as separate OBJ files for the creation of the interactive 3D pdf document (see Supporting Information), following an approach outlined in Lautenschlager (2013a) using Adobe 3d reviewer.

## Results

### Adductor musculature of the jaw

#### m. pterygoideus dorsalis (m. PTd)

**Figure 3:**
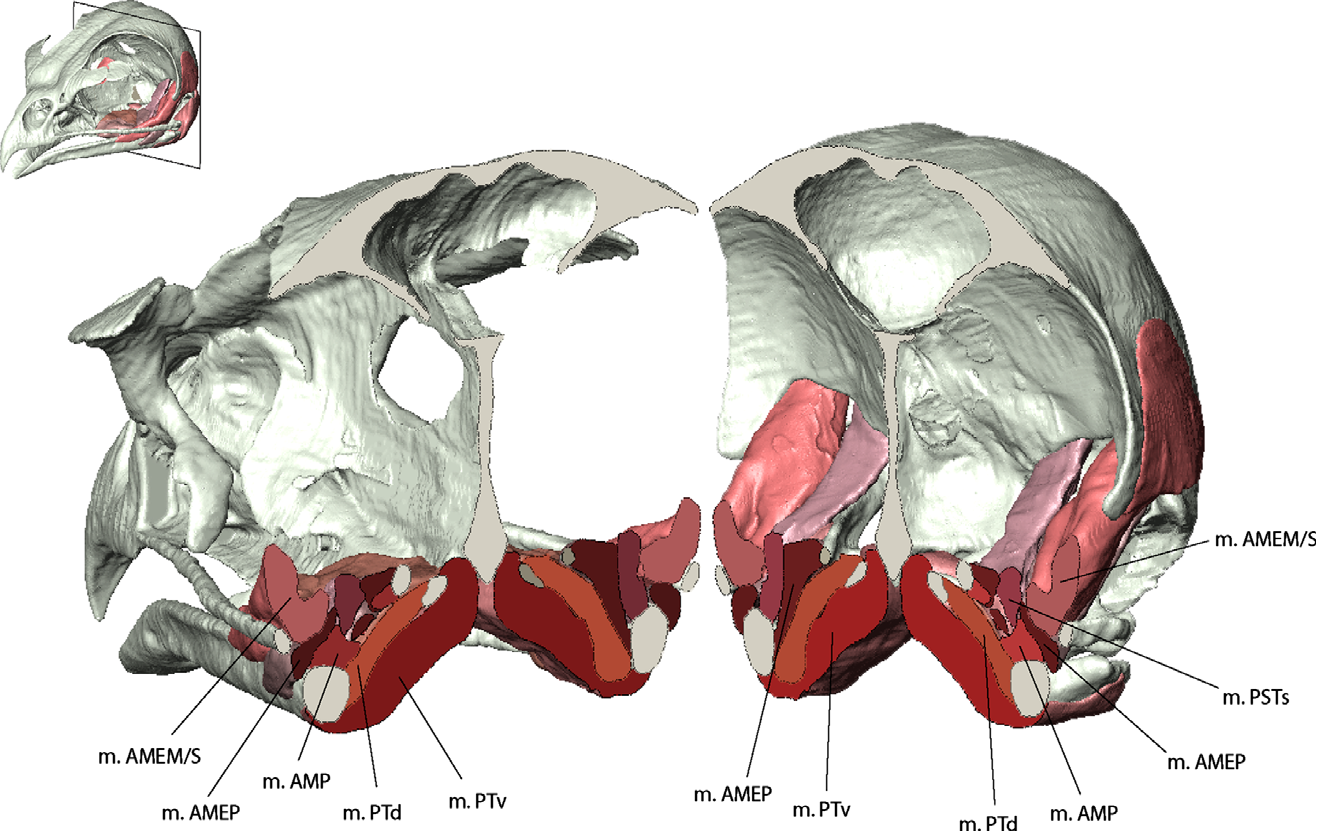
Transverse section through the skull and adductor muscle complex of *Buteo buteo*.

**Figure 4:**
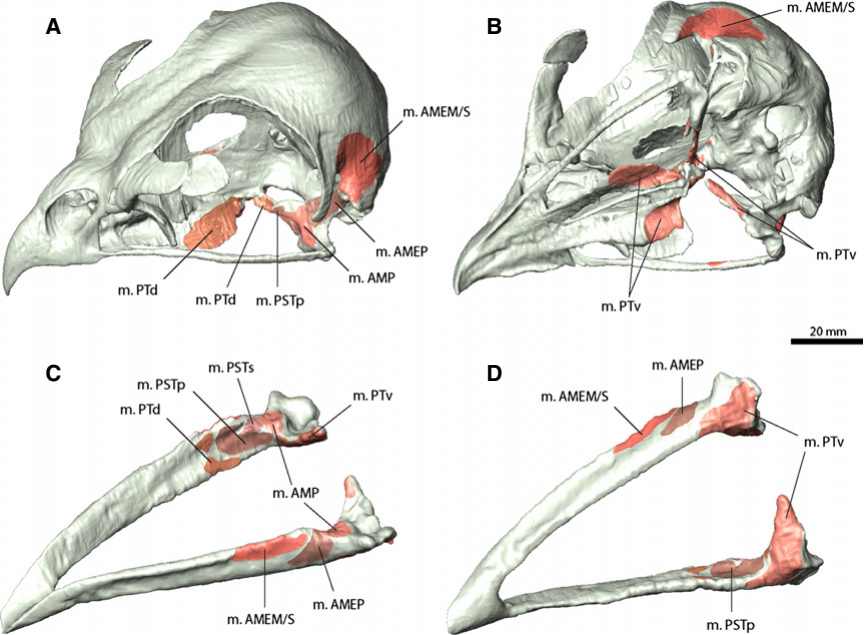
Muscle attachment sites of jaw adductor musculature of *Buteo buteo*. Muscle origins on the skull in (A) dorsolateral and (B) ventrolateral view. Muscle insertions on the lower jaw in (C) dorsolateral and (D) ventrolateral view.

**Figure 5:**
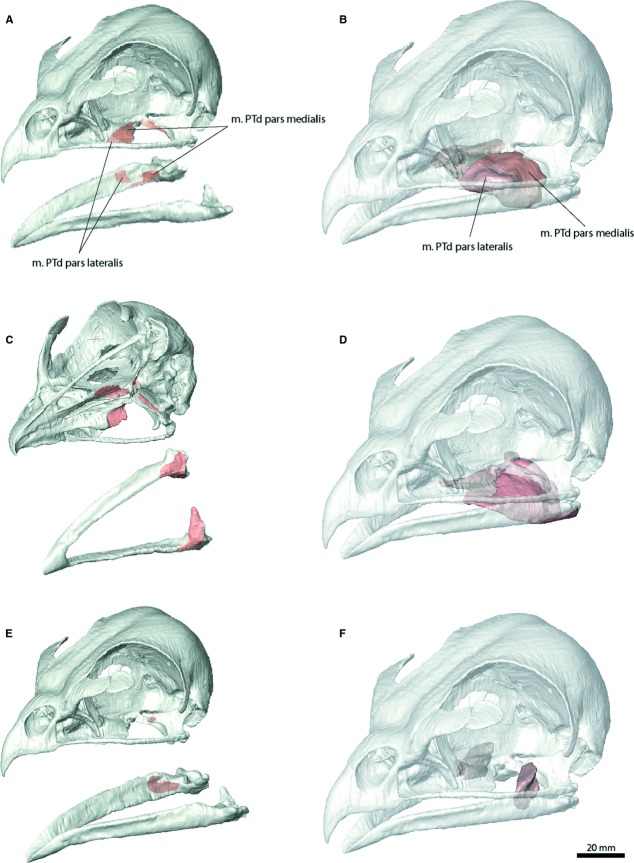
Individual adductor muscles of *Buteo buteo* with attachment sites on the skull and lower jaw (on the left) and muscle *in situ* (on the right). (A,B) m. PTd, (C,D) m. PTv, (E,F) m PSTp.

The m. pterygoideus dorsalis forms the dorsal part of the pterygoideus muscle group. Various subdivisions have been described and proposed for the m. PTd and the m. pterygoideus ventralis (m. PTv) (Lakjer, 1926; Vanden Berge & Zweers, 1993) due to the complexity of these muscles. In *Buteo buteo*, as in most other birds (Zweers, 1974; Donatelli, 2012) but in contrast to observations in *Buteo rufinus* (Onuk & Kabak, 2012), the m. PTd is subdivided into two different parts: the m. PTd pars lateralis and pars medialis. This subdivision can be observed in the CT scans, although the separation between the two tightly interwoven portions of the muscle is not always clear, which resulted in a slightly asymmetric topology in the segmented model. The m. PTd pars medialis forms the larger portion of the muscle. It originates from the dorsal surface of the palatine shelf and along the entire rostral surface of the pterygoid (Figs 3, 4A and 5A). A further origin from the interorbital septum, forming the ethmomandibularis muscle as in psittaciform birds (Holliday & Witmer, 2007), is not observed in *Buteo buteo*. The m. PTd pars medialis inserts into a depression on the caudomedial surface of the mandible, rostroventral to the jaw joint and the medial mandibular process (Figs 4C and 5A).

The m. PTd pars lateralis originates rostral to its medial counterpart from the dorsal surface of the palatine shelf (Fig. 5A). In comparison with the medial portion, the attachment area of the m. PTd pars lateralis on the palatine is only small, and likely represents a fusion with the muscle fibres with the m. PTd pars medialis, as in other birds (Donatelli, 2012). On the mandible the m. PTd pars lateralis inserts rostral to the pars medialis on the medial and ventromedial surfaces of the articular and the prearticular (Fig. 5A).

Although the concave dorsal surface of the palatal shelf clearly indicates the origin of the mPTd, there are no distinct osteological correlates marking the rostral extent or the subdivision of the muscle. On the mandible, only the insertion of the pars medialis is indicated by a shallow depression.

#### m. pterygoideus ventralis (m. PTv)

The m. pterygoideus ventralis is large and well developed in *Buteo buteo*, similar to many frugivorous pigeons and psittaciform birds (Bhattacharyya, 2013). As with the m. PTd, the m. PTv is usually subdivided into a pars medialis and a pars lateralis (Lakjer, 1926; Vanden Berge & Zweers, 1993). In *Buteo buteo*, this subdivision is difficult to differentiate. Proximally, individual attachment sites of the m. PTv can be identified (Fig. 4B). The largest portion of the muscle originates from the ventral and caudoventral surface of the palatine shelf. A further portion arises from the caudoventral surface of the pterygoid. The fibres from both attachment sites converge distally to insert on the ventral surface of the medial mandibular process, medial and caudal to the insertion of the m. PTd pars medialis (Figs 4D and 5C). Several fibres wrap around the ventral rim of the articular to attach additionally on the lateral surface of the mandible (Figs 3 and 5D), characteristic of many clades within Neoaves, including Strigiformes and Psittaciformes (Holliday & Witmer, 2007). A separate smaller belly of the m. PTv also originates from the lateroventral surface of the parasphenoid near the parasphenoid/basisphenoid contact and attaches to the dorsal tip of the medial mandibular process.

Osteological correlates for the origin of the m. PTv are present in the form of a pronounced concavity or fossa on the ventral surface of the palatine shelf. The presence and morphology of this fossa have been correlated with the development and performance of the m. PTv (Bock, 1964; van Gennip, 1986). Distally, a depression on the lateral and partly the ventral surface of the mandible below the jaw joint marks the presence of the insertion site on the bone.

#### m. pseudotemporalis profundus (m. PSTp)

The m. pseudotemporalis profundus forms the deepest muscle of the internal mandibular adductor system. It originates from the rostrolateral surface of the orbital process of the quadrate. The origination area is small and restricted to the tip of the orbital process (Figs 4A and 5E). Osteological correlates for the muscle origin, such as a marked depression on the lateral surface of the orbital process (Zusi & Bentz, 1984), are not developed in *Buteo buteo*. Along its distal course the m. PSTp is closely associated with the m. PTd, but readily separable from the latter (Fig. 3).

The m. PSTp inserts on the medial surface of the mandibular fossa, immediately rostral to the processus pseudotemporalis (tuberculum pseudotemporale after Baumel & Witmer, 1993) and ventral to the coronoid process (Figs 4C,D and 5E). It is bordered rostroventrally by the insertion of the m. PTd pars lateralis and caudally by the insertion of the m. PTd pars medialis. The mandibular branch of the trigeminal nerve passes dorsal to the m. PSTp attachment site before penetrating the bone.

#### m. pseudotemporalis superficialis (m. PSTs)

**Figure 6:**
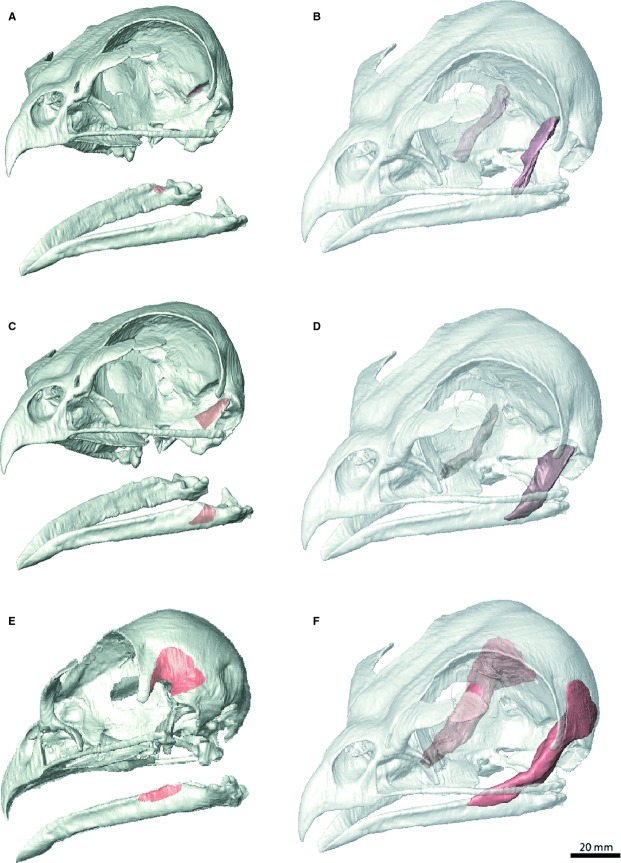
Individual adductor muscles of *Buteo buteo* with attachment sites on the skull and lower jaw (on the left) and muscle *in situ* (on the right). (A,B) m. PSTs, (C,D) m. AMEP, (E,F) m. AMEM/S.

The morphology and origin of the m. pseudotemporalis superficialis is highly variable across different bird clades (Holliday & Witmer, 2007). In *Buteo buteo*, this muscle is long, slender, and unbranched (Figs 3 and 6B), in contrast to charadriiform (Zusi, 1962), columbiform (Bhattacharyya, 2013) or galliform (Zweers, 1974) birds. The m. PSTs originates from the ventral edge of the laterosphenoid buttress in the temporal region dorsolateral to the trigeminal nerve foramen (Fig. 6A). This is evident on the bone in the form of a pronounced ridge on the rostral edge of the laterosphenoid buttress, demarcating the rostral extent of the muscle. The m. PSTs does not extend onto the caudal wall of the orbit as in other birds (Holliday & Witmer, 2007), due to the large size of the eyes, which occupy most of the orbital cavity.

Distally, the m. PSTs inserts on the processus pseudotemporalis and the caudomedial surface of the coronoid process (Figs 4C and 6A). The m. PSTs extends laterally and caudally to the m. PSTp. The mandibular branch of the trigeminal nerve originates medial to the m. PSTs proximally, passing ventral to it and then parallels the muscle laterally along most of its length, thus separating the internal adductor muscle group (m. PTd, m. PTv, m. PSTp, m. PSTs) from the external adductor muscle group (m. AMEP, m. AMEM/S) (Fig. 2B).

#### m. adductor mandibulae externus profundus (m. AMEP)

The m. adductor mandibulae externus profundus (m. adductor mandibulae externus caudalis, Vanden Berge & Zweers, 1993) is the smallest and deepest muscle of the externus (m. AME) group (Figs 3 and 6C,D). It can be clearly distinguished from the m. adductor mandibulae externus medialis/superficialis in the CT scans. The m. AMEP originates from the rostral surface of the main body and the otic process of the quadrate dorsolateral to the attachment site of the m. adductor mandibulae posterior (m. AMP) (Figs 4A and 6C). A subdivision of the m. AMEP into a pars lateralis and pars medialis, with an additional belly attaching to the laterosphenoid as found in woodpeckers (Donatelli, 2012), is not present in *Buteo buteo*.

The m. AMEP is elongate and strap-like. It passes the jugal medially and inserts along the lateral side of the mandible, rostral to the jaw joint and the insertion of the m. PTv (Figs 4D and 6C). Both the muscle origin and insertion are indistinct, leaving no traceable correlates on the bony structures.

#### m. adductor mandibulae externus medialis/superficialis (m. AMEM/S)

The lateral divisions of the externus (m. AME) muscle group have a complex morphological distribution across the different bird clades and have been variously classified and subdivided (e. g., Vanden Berge & Zweers, 1993; Zusi & Bentz, 1984; Donatelli, 2012). Amongst others, Holliday & Witmer (2007) divided the lateral portions of this muscle group topologically into a medial (m. adductor mandibulae externus medialis) and superficial (m. adductor mandibulae externus superficialis) part. In *Buteo buteo*, and generally all Neornithes, the m. AMEM and m. AMES are not distinctly separable (see Holliday & Witmer, 2007 for detailed discussion). The muscle occupies the entire temporal fossa (Figs 3 and 6F) with origins from the caudal surface of the postorbital process, the lateral surface of the squamosal, and the zygomatic process (Figs 4A,B and 6E). An additional origin of the m. AMEM/S on the quadrate body as in some galliform and anseriform birds (Hofer, 1950; Holliday & Witmer, 2007) is not present in *Buteo buteo*.

The muscle is long and extends rostrally and dorsolateral to the m. AMEP. Distally, the m. AMEM/S inserts along the dorsolateral surface of the coronoid process, rostrodorsal to the attachment site of the m. AMEP (Figs 4C,D and 6E).

#### m. adductor mandibulae posterior (m. AMP)

**Figure 7:**
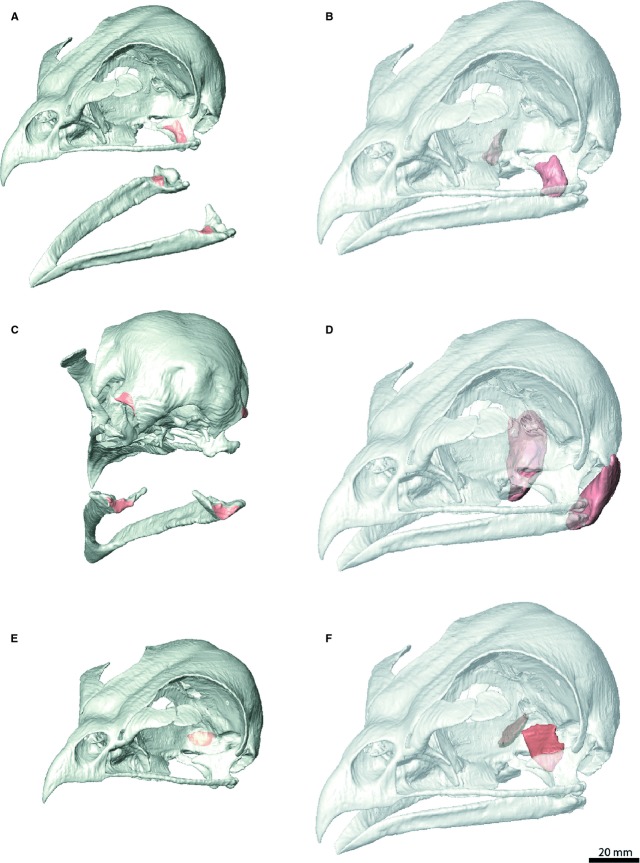
Individual adductor, depressor and protractor muscles of *Buteo buteo* with attachment sites on the skull and lower jaw (on the left) and muscle *in situ* (on the right). (A,B) m. AMP, (C,D) m. DM, (E,F) m. PPQ.

The m. adductor mandibulae posterior (m. adductor mandibulae ossi quadrati, Vanden Berge & Zweers, 1993; m. adductor mandibulae caudalis, Bühler, 1981) is located between, and partly ventral to, the internus (m. AMI) and externus (m. AME) muscle groups (Fig. 3). It originates from the ventral portion of the quadrate, encompassing most of the rostral surface of the otic and mandibular processes (Figs 4A and 7A). Osteological correlates of the muscle attachment on the quadrate are not present. It is bordered by the attachment sites of the m. PSTp medially and the m. AMEP dorsally. The m. AMP passes ventral to the m. PSTs, the m. AMEP and the m. AMEM/S, as well as the mandibular branch of the trigeminal nerve. The muscle is short and consists of a single belly only (Fig. 7B).

The m. AMP inserts on the dorsal to dorsomedial surface of the mandible, caudal to the coronoid process, lateral to the processus pseudotemporalis, and rostral to the jaw joint (Figs 4C and 7A). It is bordered by the attachments of the m. PTd pars medialis ventrally and the m. PSTs rostrally. The insertion is marked by a shallow fossa on the bone.

### Protractor and depressor musculature of the jaw

#### m. depressor mandibulae (m. DM)

The m. depressor mandibulae is a large fusiform muscle in *Buteo buteo* (Fig. 7D). As in other buzzards (Onuk & Kabak, 2012), but unlike in many other birds (Zweers, 1974; Zusi & Bentz, 1984; Donatelli, 2012), it is not subdivided into a superficial and deep portion. It is unipennate, with the individual fibres orientated along the long axis of the muscle. The muscle originates from a thin attachment site along the ventral margin and partly from the laterocaudal surface of the paroccipital process (Fig. 7C).

The m. DM inserts on the caudal surface of the articular into the prominent fossa caudalis between the small retroarticular process laterally and the medial mandibular process medially (Fig. 7C). The insertion is at an obtuse angle towards the long axis of the mandible, with the muscle directed caudodorsally toward the proximal origin. This results in a mainly caudal force transmission of the mandible and the quadrate (Bock, 1964; Zusi, 1967) during muscle contraction.

#### m. protractor pterygoidei et quadrati (m. PPQ)

The m. protractor pterygoidei et quadrati has a very variable morphology across different bird groups and has been described to consist of either two separate muscles (Lakjer, 1926) or a single muscle with subdivided insertion sites on the pterygoid and the quadrate (Vanden Berge & Zweers, 1993). In *Buteo buteo* the m. PPQ consists of a single muscle body originating from the rostromedial wall of the basisphenoid and the caudoventral corner of the orbital septum (Fig. 7E). The attachment is broad and covers the bone surface ventral to the foramen of the optic nerve and medial to the trigeminal nerve foramen.

The m. PPQ is short and stout and converges distally. It inserts along a broad attachment on the caudal surface of the orbital process of the quadrate and partly on the base of the quadrate (Fig. 7E,F). The insertion on the pterygoid is indistinct and restricted to a small area near the pterygoid/quadrate contact.

### Orbital musculature

**Figure 8:**
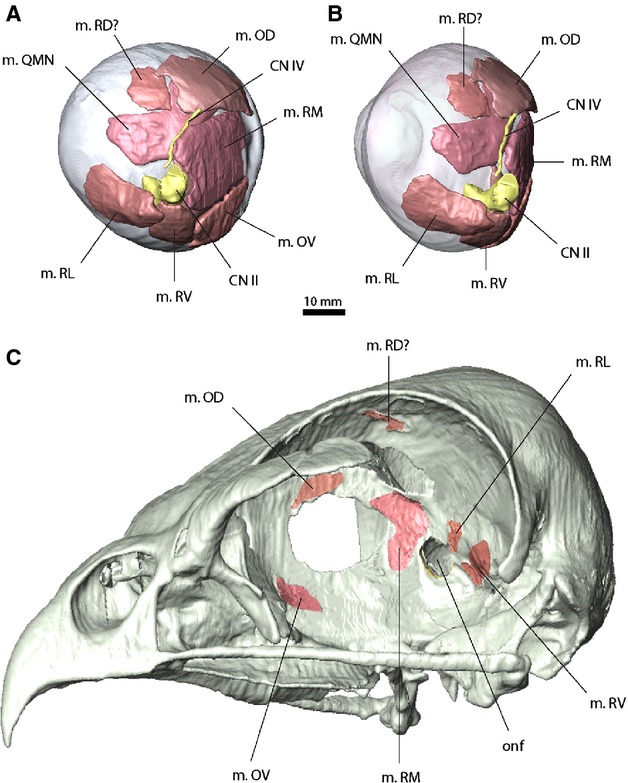
Orbital musculature of *Buteo buteo*. Left eye with musculature in (A) medial and (B) caudomedial view. (C) Cranial attachment sites of the individual muscles.

The eye musculature (musculis bulbi oculi) typically consists of six extrinsic muscles, composed of four straight and two oblique muscles (Fig. 8), acting as three antagonistic pairs. In addition, two intrinsic muscles are present in birds to control the nictitating membrane between the eyelids. The individual muscles are wrapped tightly around the eyeball and are often considerably reduced in size in most birds (Schwab, 2003; Jones et al. 2007; Jezler et al. 2010) to accommodate both the musculature and the birds' characteristically enlarged eyeballs in the orbital cavity.

#### m. rectus lateralis (m. RL)

The m. rectus lateralis originates from a small, shallow depression on the caudal wall of the orbital cavity, lateral to the foramen for the optic nerve and dorsal to the attachment of the m. PPQ (Fig. 8C). The m. RL fans out distally to insert caudally on the medial to ventromedial rim of the eyeball, passing the optic nerve (CN II) laterally (Fig. 8A,B).

#### m. rectus medialis (m. RM)

The m. rectus medialis is a large and flattened muscle originating from the interorbital septum, rostromedial to the optic nerve foramen (Fig. 8C). Distally, the muscle inserts on the medial to rostromedial rim of the eye (Fig. 8A,B). In comparison with the other eye muscles, the m. RM is large and well-developed.

#### m. rectus ventralis (m. RV)

The m. rectus ventralis is a small muscle originating from the orbital wall, with the attachment located ventral to the origin of the m. RL and lateral to the optic nerve foramen (Fig. 8C). The m. RV passes between the m. RL and the m. obliquus ventralis (m. OV) and inserts on the ventral rim of the eyeball (Fig. 8A,B).

#### m. rectus dorsalis (m. RD)

The m. rectus dorsalis could not be clearly identified in *Buteo buteo*. A possible structure representing this muscle originates from the dorsal surface of the orbital cavity (Fig. 8C) and inserts on the mediodorsal rim of the eye. In comparison with other birds (Zusi & Bentz, 1984; Vanden Berge & Zweers, 1993) the origin of this muscle and the reduced size are both unusual. As in Strigiformes (Schwab, 2003), the m. RD might be considerably atrophied in *Buteo buteo*.

#### m. obliquus ventralis (m. OV)

The m. obliquus ventralis originates from the rostroventral surface of the mesethmoid region of the interorbital septum (Fig. 8C). The m. OV passes the m. RV rostrally and inserts on the rostroventral rim of the eyeball ventral to the m. RM (Fig. 8A,B).

#### m. obliquus dorsalis (m. OD)

The m. obliquus dorsalis forms the dorsal counterpart to the m. OV. It originates from the dorsal surface of the interorbital septum above the large fonticulus interorbitalis (Fig. 8C). The m. OD inserts along an extended attachment site on the dorsomedial to dorsorostral rim of the eye (Fig. 8A,B). The m. OD is innervated by the trochlear nerve (CN IV) and the contact between the muscle and the nerve is clearly resolved in the CT scans.

#### m. quadrates membrane nictitantis (m. QMN)

The m. quadrates membrane nictitantis is a large muscle covering the medial surface of the eyeball ventral to the m. OD (and the possible m. RD) and dorsal to the optic nerve (Fig. 8A). It is largely covered by the m. RM. and m. OD rostrally. Starting at the origin near the optic nerve, the m. QMN fans out dorsally.

#### m. pyramidalis membrane nictitantis (m. PMN)

The m. pyramidalis membrane nictitantis forms the ventral counterpart to the m. QMN. In comparison with the latter, the m. PMN is considerably smaller. It originates from the ventral rim of the eyeball between the insertions of the m. RV and the m. OV. Similar to the m. QMN, it fans out dorsally, but to a lesser degree.

### Hyoid musculature

#### m. serpihyoideus (m. SE)

**Figure 9:**
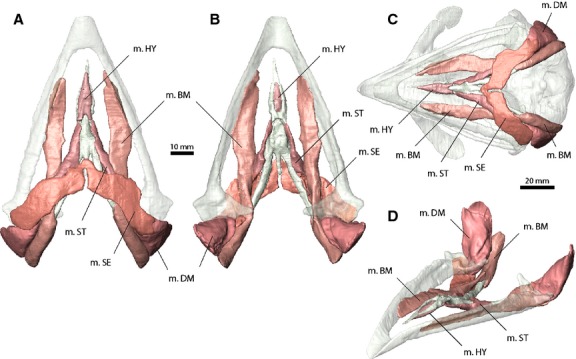
Hyoid musculature of *Buteo buteo* in (A,C) ventral, (B) dorsal and (D) rostrolateral view.

The m. serpihyoideus and m. stylohyoideus (m. ST) were difficult to differentiate in the specimen of *Buteo buteo* as both muscles are closely conjoined. The m. SE is a large, flat and strap-like muscle, which originates from the dorsolateral surface of the articular and the retroarticular process (Fig. 9A,B). The muscle arches rostromedially, overlying the m. PTv and partly the distalmost portion of the m. DM, and more medially the m. branchiomandibularis (m. BM). Distally, the m. SE inserts along the lateral and ventral surfaces of the caudal part of the basibranchiale caudale (= urohyale *sensu* Baumel & Witmer, 1993).

#### m. stylohyoideus (m. ST)

The m. stylohyoideus arises rostrally from the m. SE (Fig. 9A), at which point both muscles are tightly interwoven. Due to the close association, however, it is not clear exactly where the m. ST originates. The m. ST runs rostrally parallel to the hyoid bones and inserts on the lateral surface of the basibranchiale rostrale (= basihyale *sensu* Baumel & Witmer, 1993).

#### m. branchiomandibularis (m. BM)

The m. branchiomandibularis forms the largest muscle of the hyoid muscle complex. It originates from an elongate attachment along the medial surface of the dentary (Fig. 9A,D). The muscle overlies the m. SE, the m. ST and the ceratobranchiale. Caudally, at the level of the jaw joint, the muscle forms a medially opening trough-like structure, which wraps around the caudal portion of the ceratobranchiale and the epibranchiale. Caudal to this attachment point, the m. BM completely covers the epibranchiale for most of its length. The epibranchiale and thus the caudal portion of the m. BM. curve dorsally around the caudal (= external) surface of the DM (Fig. 9C,D).

#### m. hypoglossus (m. HY)

The m. hypoglossus of birds can usually be subdivided into a pars rostralis and a pars obliquus (Vanden Berge & Zweers, 1993; Huang et al. 1999) but this differentiation is not clear in the studied specimen of *Buteo buteo*. The muscle is small and restricted to the rostral portion of the hyoid (Fig. 9A,B). It originates from the ventral surface of the basibranchiale rostrale and inserts on the ventral surface of the entoglossum (= paraglossum *sensu* Baumel & Witmer, 1993).

### Cervical musculature

**Figure 10:**
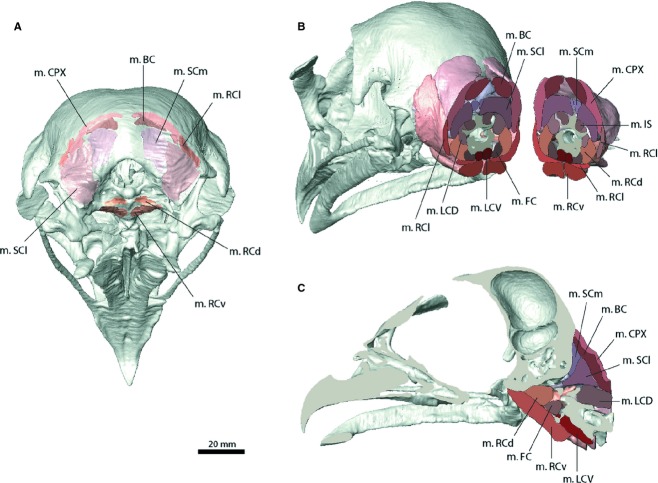
Cervical musculature of *Buteo buteo* with (A) cranial attachment sites in caudoventral view, (B) transverse and (C) sagittal section through the neck and skull.

The majority of the neck muscles attaching to the back of the skull were identified and visualised (Fig. 10). As the head of the specimen was severed at the fourth cervical vertebra, not all of the vertebral origins of the individual muscles were preserved. Thus, only the rostralmost parts of the craniocervical musculature are described. The same applies to the inter-vertebral musculature.

#### m. complexus (m. CPX)

**Figure 11:**
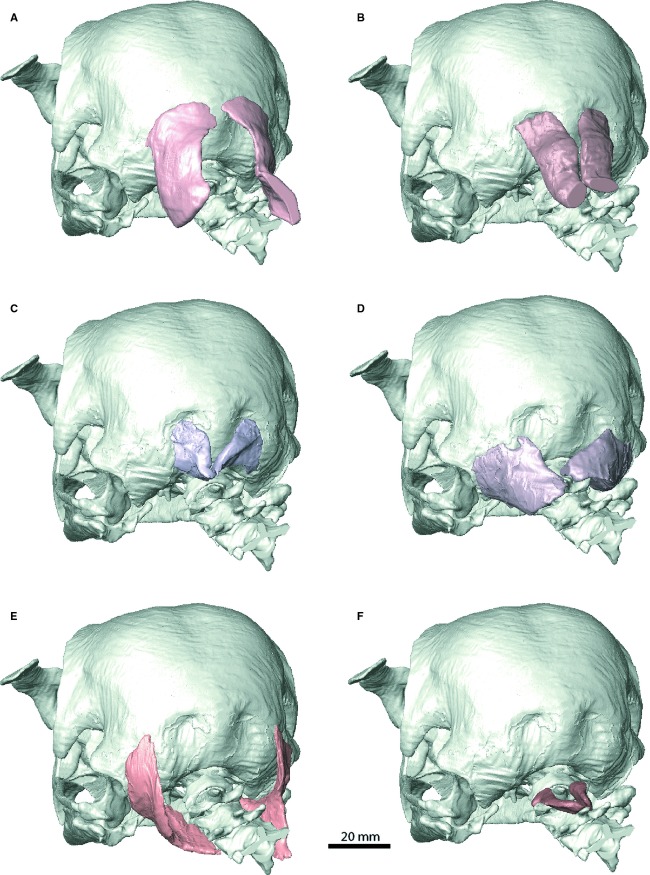
Individual cervical muscles of *Buteo buteo* in caudal view. (A) m. CPX, (B) m. BC, (C) m SCm, (D) m. SCl, (E) m. RCl, (F) m. RCd.

The m. complexus is the most superficial muscle of the craniocervical muscle complex. The muscle is broad and strap-like and covers the deeper musculature laterodorsally (Figs 10B,C and 11A). The m. CPX usually originates from the transverse processes of cervical vertebrae (CV) 3–5 (Zusi & Bentz, 1984; Landolt & Zweers, 1985; Snively & Russell, 2007) and/or the diaphophyses of CV4–CV6 (Jenni, 1981). In the studied specimen of *Buteo buteo*, the muscle did not originate from the preserved vertebrae, indicating that the origin of m. CPX lies caudal to CV 4.

The m. CPX inserts on the parietals on each side of the nuchal crest. The insertion extends laterally to the squamosal contact, forming a lunate attachment site (Fig. 10A).

#### m. biventer cervicis (m. BC)

In most birds the m. biventer cervicis is separated into two distinct parts or bellies connected by a long tendon (Vanden Berge & Zweers, 1993). The caudal belly usually originates from the caudal cervical vertebrae at the base of the neck (Landolt & Zweers, 1985; Snively & Russell, 2007) and forms the larger part of the muscle. It ends rostrally in the connecting tendon, which covers the region between CV3 and CV9 (Jenni, 1981; Zusi & Bentz, 1984; Snively & Russell, 2007), depending on the taxon. The rostral part of m. BC arises from the connecting tendon, but this region is not present in the studied specimen and must have occurred caudal to CV4. As preserved, the rostral part of the m. BC is a short and stout muscle (Fig. 11B). It inserts on the parietals immediately ventral to the insertion of m. CPX and lateral to the nuchal crest (Fig. 10A,B).

#### m. splenius capitis (m. SC)

The m. splenius capitis has a very variable morphology in different groups of birds (Burton, 1971; Brause et al. 2009) and is sometimes subdivided into a medial (m. SCm) and lateral (m. SCl) part (Snively & Russell, 2007). In *Buteo buteo*, this subdivision is prominent and clearly recognisable in the CT scans. Both parts originate from the rostrodorsal surface of the neural spine of CV2, with the medial part lying deep to its lateral counterpart (Fig. 10B). An interdigitating cruciform pattern or extension onto the third cervical vertebra as seen in hummingbirds and other bird families (Burton, 1971; Zusi & Bentz, 1984) is absent in *Buteo buteo*.

Both subdivisions of the m. SC increase in size proximally and fan out to insert along the basicranial surface dorsal and lateral to the foramen magnum (Fig. 10A). The insertion of the medial part of the muscle lies to either side of the nuchal crest on the parietals and the supraoccipital, ventral to the insertion of the m. BC. The lateral part of the m. SC inserts on the paroccipital process, ventral to the attachment of the m. rectus capitis lateralis (m. RCl) and lateroventral to the attachment of the m. CPX.

#### m. rectus capitis lateralis (m. RCl)

The m. rectus capitis lateralis lies lateral to the m. rectus capitis dorsalis (m. RCd) and the m. SCl and the internal carotid artery (Fig. 10B). The muscle originates from the ventral processes of CV2 and CV3, but might have further origins from CV4 and CV5 and from the m. longus colli dorsalis pars profunda, as in other birds (Jenni, 1981; Snively & Russell, 2007).

The m. RCl wraps laterally around the neck, forming a thin and flattened muscle, and inserts on the lateral rim of the paroccipital process (Figs 10B and 11E). The attachment site is elongate and crescent-shaped and located ventral to the insertion of m. CPX and lateral to the insertion of m. SCl.

#### m. rectus capitis dorsalis (m. RCd)

The m. rectus capitis dorsalis is the deepest muscle of the rectus capitis muscle complex (Fig. 10B). As in other birds (Jenni, 1981; Zusi & Bentz, 1984), it originates from the lateral surface of the atlas (C1) and the lateral to lateroventral surfaces of the transverse processes of CV2–CV4 in *Buteo buteo* (Fig. 11F). Whether the muscle has further origins from more caudally located vertebrae (Landolt & Zweers, 1985; Snively & Russell, 2007) is not clear. The individual muscle slips merge ventrally into a stout muscle belly.

The m. RCd inserts on the basioccipital rostral to the occipital condyle and the foramina for cranial nerves X and XII (Fig. 10A). In comparison with other taxa (Snively & Russell, 2007), the attachment is unobtrusive, without any prominent ridges or tubercles on the basioccipital surface.

#### m. rectus capitis ventralis (m. RCv)

**Figure 12:**
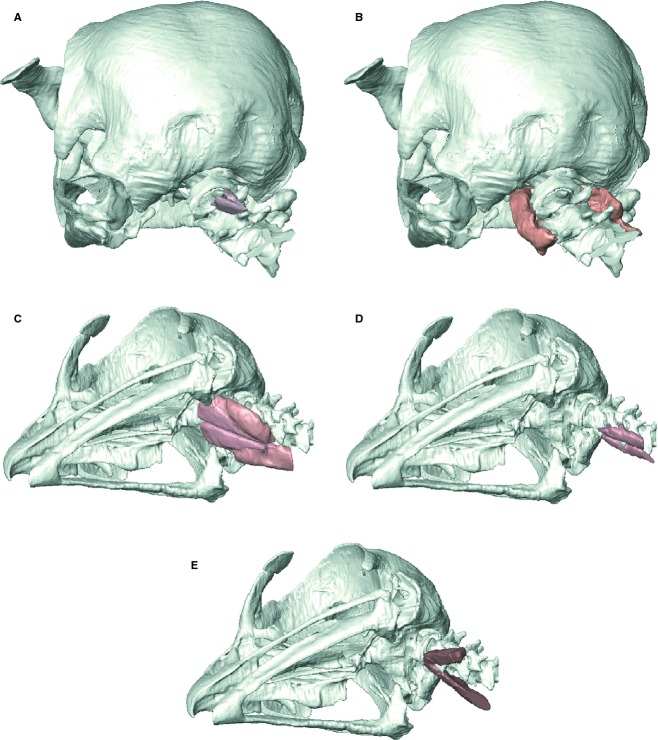
Individual cervical muscles of *Buteo buteo* in (A,B) caudal and (C-E) lateroventral view. (A) m. IS, (B) m. LCD, (C) m RCv, (D) m. LCV, (E) m. FC.

The m. rectus capitis ventralis can be subdivided into a pars medialis and a pars lateralis (Fig. 12C). Both parts are arranged parallel to each other and become closely associated rostrally near the basicranial insertion (Landolt & Zweers, 1985). The pars medialis originates from the ventral processes of CV2 and CV3, the pars lateralis, and possibly from the ventrolateral surface of the atlas, but the attachment at this point is not resolved clearly enough in the CT scans. The pars lateralis originates from the ventral processes of CV3 and CV4 and, as is the case in other birds, most likely also from CV5 and CV6 (Jenni, 1981; Landolt & Zweers, 1985; Snively & Russell, 2007), but this region is not preserved in the studied specimen. As the name implies, the pars lateralis parallels its medial counterpart laterally.

The pars medialis and lateralis merge at the level of CV3 and both parts insert rostral to the attachment of the m. RCd on the basitemporal plate (Fig. 10A,C).

#### m. longus colli dorsalis (m. LCD)

The m. longus colli dorsalis is a complicated system of muscles situated at the dorsal part of the neck (Figs 10B,C and 12B). It is variously subdivided into up to four different parts: pars cranialis, pars caudalis, pars profunda and sometimes pars thoracica (Landolt & Zweers, 1985 and references therein; Vanden Berge & Zweers, 1993). In the studied specimen of *Buteo buteo* only the rostralmost part of pars cranialis is preserved. The pars cranialis can consist of a variable number of muscle bellies (Zusi & Bentz, 1984; Landolt & Zweers, 1985), the rostral of which originates from the lateral surface of the neural spine of CV3. It inserts on the neural arch of the axis (CV2) and partly on that of the atlas (CV1).

#### m. longus colli ventralis (m. LCV)

The m. longus colli ventralis is a large muscle located on the ventral side of the vertebral column (Fig. 10B,C). It consists of a series of overlapping muscle bellies, connecting the rostralmost cervical vertebrae to the notarial vertebrae. In most birds, the m. LCV can be subdivided into a pars cranialis and a pars caudalis (Landolt & Zweers, 1985), but in the specimen of *Buteo buteo* only the rostralmost part of the pars cranialis is preserved.

The single bellies constituting this muscle each originate from the carotid processes on the ventral surface of the vertebrae and insert on the costal processes of the preceding vertebra.

#### m. flexor colli (m. FC)

The m. flexor colli is sometimes subdivided into a medial and lateral part (Vanden Berge & Zweers, 1993), but this differentiation is not recognisable in *Buteo buteo*. The muscle is located on the lateroventral part of the neck and lies lateral to the m. LCV (Figs 10B and 12E). It originates from the transverse process of CV3, and most likely CV4 and CV5 (Zusi & Bentz, 1984; Landolt & Zweers, 1985), but this region was not complete enough to identify the additional origins clearly. Proximally, the m. FC converges towards the insertion on the costal process of the atlas (CV1).

#### m. interspinales (m. IS)

The m. interspinales can be clearly identified in *Buteo buteo*. The m. IS consists of a series of small muscles connecting adjacent vertebrae (Fig. 12A). The muscle complex lies deep to the m. LCD pars cranialis (Fig. 10B). In the studied specimen, the muscle originates from the rostrodorsal surface of the neural spine and inserts on the caudodorsal to lateral surface of the neural spine of the vertebra rostral to its origin.

### Ligaments

#### Ligamentum postorbitale (l. PO)

**Figure 13:**
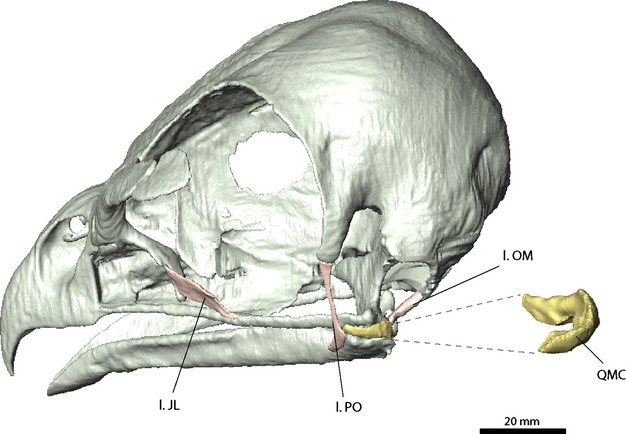
Ligaments of *Buteo buteo in situ* in left lateral view, with quadrate-mandibular cartilage enlarged.

The ligamentum postorbitale (Fig. 13) is assumed to play a functional role in the mechanical coupling (Nuijens et al. 2000) and the coordination of the upper and lower jaw (Zusi, 1967). In *Buteo buteo* the ligamentum postorbitale is long and thin. Dorsally, it attaches to the postorbital process of the laterosphenoid. The ligament extends ventrally and crosses the caudal part of the jugal laterally. It attaches along the rostral edge of the lateral mandibular process on the mandible.

#### Ligamentum occipito-mandibulare (l. OM)

The ligamentum occipito-mandibulare is a short but stout ligament connecting the cranium to the articular region of the mandible caudal to the jaw joint (Fig. 13). The ligament lies medial to the m. DM. It attaches to the tip of the paroccipital process and extends rostroventrally, where it attaches to the caudal surface of the retroarticular process.

#### Ligamentum jugolacrimale (l. JL)

The ligamentum jugo-lacrimale is thin and strap-like (Fig. 13). It originates from the tip and the lateral surface of the lacrimal. The ligament extends caudoventrally and inserts on the dorsolateral surface of the jugal.

The ligamentum suborbitale (l. SO) and lacrimomanibulare (l. LM), which also originate from the lacrimal, could not be identified in the specimen of *Buteo buteo*. The l. LM is characteristic of Anseriformes (Baumel & Raikow, 1993) and might be absent in *Buteo buteo*.

#### Quadrato-mandibular cartilage (QMC)

The caudal cartilage cap between the quadrate-mandibular articulation on the jaw joint could be clearly identified and visualised in *Buteo buteo* (Fig. 13). It is crescent-shaped and closely follows the caudal articular margin. In cross-section the cartilage is wedge-shaped, which limits the degree of opening of the lower jaw.

### Endocranial anatomy

In contrast to the musculature, the iodine staining did not significantly enhance the contrast of all endocranial tissues, in particular the brain. For other structures such as the inner ear, the exposure to the iodine solution resulted in a shrinkage of tissues. Thus the described anatomy is based largely on the digital endocast of the brain and endosseous labyrinth. Neurovascular structures were well-resolved.

#### Brain

**Figure 14:**
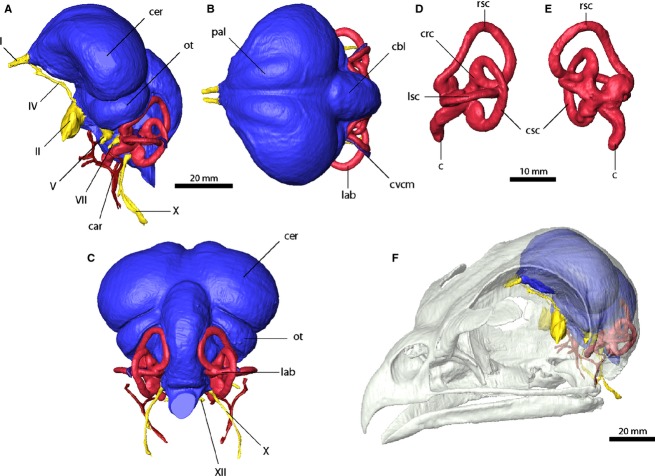
Endocranial anatomy of *Buteo buteo* in (A) left lateral, (B) dorsal and (C) caudal view. Left endosseous labyrinth in (D) lateral and (E) medial view. (F) Brain, endosseous labyrinth and neurovascular structures *in situ*. Brain and endosseous labyrinth based on casts of the cranial cavity.

The brain (Fig. 14A–C) is very large in *Buteo buteo* and takes up a significant portion of the skull (Fig. 14F). The individual brain portions (telencephalon, mesencephalon and metencephalon) are arranged vertically. The brain is dominated by an enlarged telencephalon, which comprises the olfactory tracts/bulbs and the cerebral hemispheres. As is characteristic for most extant birds, the olfactory tracts/bulbs are small and unobtrusive (Bang & Cobb, 1968; Zelenitsky et al. 2011). The cerebral hemispheres are prominently enlarged and extend mediolaterally. Dorsally, they are separated by a pronounced interhemispherical fissure. A distinct hyperpallium (Wulst/eminentia sagittalis) is visible lateral to the interhemispherical fissure on each hemisphere.

The mesencephalon is clearly represented by large and prominent optic tecta, located ventral to the cerebral hemispheres and separated from the telencephalon by distinct valleculae (valeculla telencephali).

The metencephalon, largely represented by the cerebellum, is only of moderate size in comparison with the other brain divisions. The cerebellum is mediolaterally thin. The floccular lobes (cerebellar auricles) are only weakly developed and very small and do not project beyond the rostral semicircular canal.

#### Neurovascular structures

The majority of the cranial nerves originating from the brain could be identified and visualised in the specimen of *Buteo buteo*. The olfactory nerves (CN I) originate from the olfactory tracts rostral to the cerebral hemispheres. They project rostrally, where they are separated by the interorbital septum. The optic nerves (CN II) are large, originating from the diencephalon rostral to the optic tecta. After exiting the braincase, the optic nerves project rostrolaterally to innervate the eyes. The oculomotor (CN III) nerve could not be identified. The trochlear nerves (CN IV) originate medially and slightly dorsal to the optic nerves. They run rostrodorsally and innervate the m. obliquus dorsalis of the eye musculature. The trigeminal nerve (CN V) is small. It projects laterally, ventral to the optic tecta. Divisions of CN V into individual branches could not be resolved in detail. As with the oculomotor nerve, the abducens nerve (CN VI) could not be identified; this is likely due to the staining agent not penetrating these structures. The facial (VII) and vestibulocochlear nerves (CN VIII) originate from the pons ventral to the floccular lobes and exit the braincase through a single shared foramen. Distally, the two nerves diverge, with the facial nerve projecting rostrally and the vestibulocochlear nerve innervating the cochlear region of the inner ear. A subdivision into the vestibular and cochlear branch could not be resolved. The vagus nerve (CN X) was clearly identified originating from the pons caudal to the vestibulocochlear nerve. It exits the braincase through a small foramen in the caudal braincase wall. The glossopharyngeal (CN IX) and accessory nerves (CN XI) could not be resolved. A single small hypoglossal nerve (CN XII) originates at either side of the medulla and exits the braincase through a foramen lateral to the occipital condyle.

Similarly to some of the cranial nerves, the staining agent did not produce sufficient contrast to distinguish all the vascular tissues so that only very large and prominent structures could be resolved and visualised. The internal carotid artery (car) originates from the pituitary region ventral to the optic nerves. It enters the braincase through the basisphenoid, branching several times to form the anastomosis with the cerebral arteries, before exiting the skull caudally. The middle cerebral vein originates in the floccular region. It passes through the rostral semicircular canal, ventral to the lateral semicircular canal and rostral to the caudal semicircular canal, and exits the braincase within the tympanic region.

#### Endosseous labyrinth

The endosseous labyrinth shows a characteristic avian morphology (Fig. 14 D,E). The individual semicircular canals are elongate. The rostral semicircular canal is the longest and has an elliptical outline, due to the rostral displacement by the optic tecta. The caudal and lateral semicircular canals approach a more circular outline. The three canals are joined at the crus communis, which is located at the centre of the rostral semicircular canal, resulting in a twisting of the other two canals. The caudal and lateral semicircular canals communicate in the typical avian condition (Gray, 1908) but a communication between the rostral and lateral semicircular canal, as in Charadriiformes (Smith & Clarke, 2012) and some Strigiformes (Witmer et al. 2008), is absent in *Buteo buteo*. The ampullae, located at the base of the individual canals, are enlarged but flattened along the plane described by the respective canals.

The cochlear duct is elongate. It projects rostrally and medially, when the endosseous labyrinth is situated with the lateral semicircular canal in a horizontal orientation.

## Discussion

As presented above, contrast-enhanced CT scanning using iodine staining produces a valuable resource to identify, visualise and document soft-tissue structures in extant specimens. One of its main advantages over traditional dissection techniques lies in the fact that specific organs and internal features can be investigated selectively and *in situ*, in relation to other soft and hard tissues. This allows the observation of the segmented structures in their true dimensions and positions, making them easily quantifiable. When applied prior to gross dissection, iodine staining can be used to provide a guideline for the physical dissection process, where digital findings may be confirmed. As a largely non-destructive method (apart from the fixing and staining of the tissues), it can be applied multiple times to the same specimen to selectively enhance the contrast of specific tissues. With the increasing importance of tomographic methods in studies of taxonomy and comparative biomechanics, iodine staining may be a valuable approach for obtaining detailed anatomical information from rare specimens or type material (Faulwetter et al. 2013).

Moreover, detailed knowledge of soft-tissue anatomy in extant taxa provides a vast amount of information, which can be applied to extinct taxa. Although soft tissues in fossils are only preserved in rare, exceptional cases (Kellner, 1996), extant phylogenetic bracketing approaches allow inferences of the presence of soft-tissue structures (and thus possibly physiology or even behaviour) in extinct animals by comparing them with their nearest living relatives (Witmer, 1995). Various soft tissues, in particular muscles or neurovascular structures, may leave identifiable traces on the bony structure, such as ridges, crests or grooves, which can be used to infer the presence of the respective soft tissues. However, accurate information on these osteological correlates and the corresponding soft-tissue structure is required. Detailed anatomical data derived from contrast-enhanced tomography can thus provide easily accessible and comprehensible resources. Extant archosaurs, namely birds and crocodilians, provide a large dataset, which can be used to bracket soft-tissue anatomy in their extinct relatives, for example non-avian dinosaurs (Holliday, 2009). Although some three-dimensional datasets on crocodilian anatomy are available (Holliday et al. 2013), none exists providing details on avian anatomy, which could help to refine the bracketed reconstruction of muscular systems in extinct animals (Lautenschlager, 2013b). In the case of *Buteo buteo* presented here, the clearest osteological correlates are found for the muscles in the adductor complex. In particular, the m. PTv, the m. PTd, the m. PSTs and the m. AMP leave discernible traces on the bone. In contrast, the external adductor muscles (m. AMEP, m. AMEM/S) and the m. PSTp show no or only faint osteological correlates, but the presence of osteological correlates, in particular for the m. PSTp, is highly variable among different bird clades (Zusi & Bentz, 1984). Other muscle groups, such as the ocular or hyoid musculature, show very few or no unambiguous correlates. Although the presented example of *Buteo buteo* provides a detailed map of the anatomical structures, the plasticity of hard-and soft-tissue correlates requires further studies across a wider range of different bird taxa.

Although iodine staining significantly increases resolution and tissue contrast, and tomographic methods have become increasingly cost-effective and comparably widely available in recent years, researchers face some limitations when working with iodine-stained datasets. For instance, it is still necessary to manually segment contrast-enhanced CT data. This often requires expensive computer hard-and software, and a considerable degree of technical expertise and user time to complete the segmentation and 3D visualisation process, posing a large obstacle to many researchers. To process the wealth of information provided by contrast-enhanced tomography in a quick and efficient manner, the automisation of individual segmentation procedures will become necessary. Similar tools are already applied in the medical sciences (Buie et al. 2007; Campadelli et al. 2009; Chung et al. 2009), and can most likely be transferred to other disciplines with little difficulty.

Furthermore, whereas some soft-tissue structures allow relatively quick and accurate quantification (e.g. muscle cross-section area or volume), other properties (pennation angles, fascicle length, aponeurotic/tendinous muscle attachments) depend variably on the resolution of the tomographic method and the spatial orientation of the specimen within the CT scanner: for example, the fascicle orientation of muscles can only be resolved when the orientation of the muscle is oblique to the spatial axes of the CT dataset. Although most of the soft tissues and organs can be resolved using iodine staining, it does not enhance the contrast for delicate structures, such as very small neurovascular structures. In this case other contrast-enhancing methods, for example vascular injection (Holliday & Witmer, 2007), can be used to supplement iodine-stained datasets.

Finally, although iodine staining has previously been assumed to be reversible (Bock & Shear, 1972), the mechanism by which the stain is taken up by tissues and the long-term effects of the staining solution on specimens are yet to be fully explored or understood. The addition of I_2_KI to a fixing solution does result in the shrinkage of certain soft tissues beyond that which would be expected from immersion in a fixing solution alone, and the degree to which this happens is primarily dependent on the I_2_KI concentration (Vickerton et al. 2013); this may impede accurate quantification of tissue volumes, as observed here in the inner ear tissues. Although work has begun to look at how differences in I_2_KI concentration, immersion time, specimen size, freezing, and CT scanner settings affect the contrast and shrinkage of tissues (Jeffery et al. 2011; Vickerton et al. 2013), further study of the long-term effects and reversibility of iodine staining on specimens is needed.

As the anatomical and morphological data derived from the CT scanning and segmentation processes consequently exist in the digital realm, it is relatively easy to adapt it for further computational analysis; for example, for geometric morphometric analysis, or to provide input parameters such as muscle forces and vectors, and skeletal geometry, for finite element analysis (Cox et al. 2011) or multi-body dynamics analysis. Furthermore, using interactive tools such as 3D pdf documents (Lautenschlager, 2013a; see also Supporting Information) or java-based applications (Ruffins et al. 2007), the segmented CT scans can be presented as high-resolution, three-dimensional, morphological datasets (Düring et al. 2013). These can be easily disseminated for comparative studies or teaching purposes to convey complicated biological structures.

## Conclusions

Contrast-enhanced tomographic scanning using iodine staining, when combined with three-dimensional visualisation techniques, provides a powerful tool for anatomical, taxonomic and functional research, and can be applied to a variety of specimens and research questions. As shown in the presented example of *Buteo buteo,* individual hard-and soft-tissue structures can be identified and investigated *in situ*. Although some limitations remain, iodine staining can be used to produce comprehensive anatomical datasets, which can subsequently be used for descriptive and illustrative purposes, and beyond in further computational analysis.

## Appendix

### Anatomical abbreviations used in figures and Supporting Information

**Table:**

c	cochlear duct
car	carotid artery canal
cer	cerebral hemisphere
cbl	cerebellum
crc	crus communis
csc	caudal semicircular canal
cvcm	caudal middle cerebral vein
endo	brain endocast
l. JL	ligamentum jugolacrimale
l. OM	ligamentum occipito-mandibulare
l. PO	ligamentum postorbitale
lab	endosseous labyrinth
lsc	lateral semicircular canal
m. AMEM/S	m. adductor mandibulae externus medialis/superficialis
m. AMEP	m. adductor mandibulae externus profundus
m. AMP	m. adductor mandibulae posterior
m. BC	m. biventer cervicis
m. BM	m. branchiomandibularis
m. CPX	m. complexus
m. DM	m. depressor mandibulae
m. FC	m. flexor colli
m. HY	m. hypoglossus
m. IS	m. interspinales
m. LCD	m. longus colli dorsalis
m. LCV	m. longus colli ventralis
m. OD	m. obliquus dorsalis
m. OV	m. obliquus ventralis
m. PPQ	m. protractor pterygoidei et quadrati
m. PSTs	m. pseudotemporalis superficialis
m. PSTp	m. pseudotemporalis profundus
m. PTd	m. pterygoideus dorsalis
m. PTv	m. pterygoideus ventralis
m. RCd	m. rectus capitis dorsalis
m. RCl	m. rectus capitis lateralis
m. RCv	m. rectus capitis ventralis
m. RD	m. rectus dorsalis
m. RL	m. rectus lateralis
m. RM	m. rectus medialis
m. RV	m. rectus ventralis
m. SCm	m. splenius capitis medialis
m. SCl	m. splenius capitis lateralis
m. SE	m. serpihyoideus
m. ST	m. stylohyoideus
onf	optic nerve foramen
ot	optic tecta
pal	pallium
QMC	quadrato-mandibular cartilage
QMN	quadrates membrane nictitantis
rsc	rostral semicircular canal
I	olfactory nerve
II	optic nerve
IV	trochlear nerve
V	trigeminal nerve
VII	facial nerve
X	vagus nerve
XII	hypoglossal nerve canal

## Supporting Information

Fig S1 Interactive 3D pdf showing the digitally segmented hardand soft-tissue structures of the Common *Buzzard Buteo buteo.*[.pdf]

Video S1 Movie file of the CT dataset showing the stained specimen in coronal section in rostrocaudal direction. [.mpeg]
