# Supplementary figures and images for: Digital dissection – using contrast-enhanced computed tomography scanning to elucidate hard-and soft-tissue anatomy in the Common Buzzard Buteo buteo
Source: J Anat. 2013 Dec 18;224(4):412–31. doi: 10.1111/joa.12153 (PMC4098676; doi:10.1111/joa.12153)

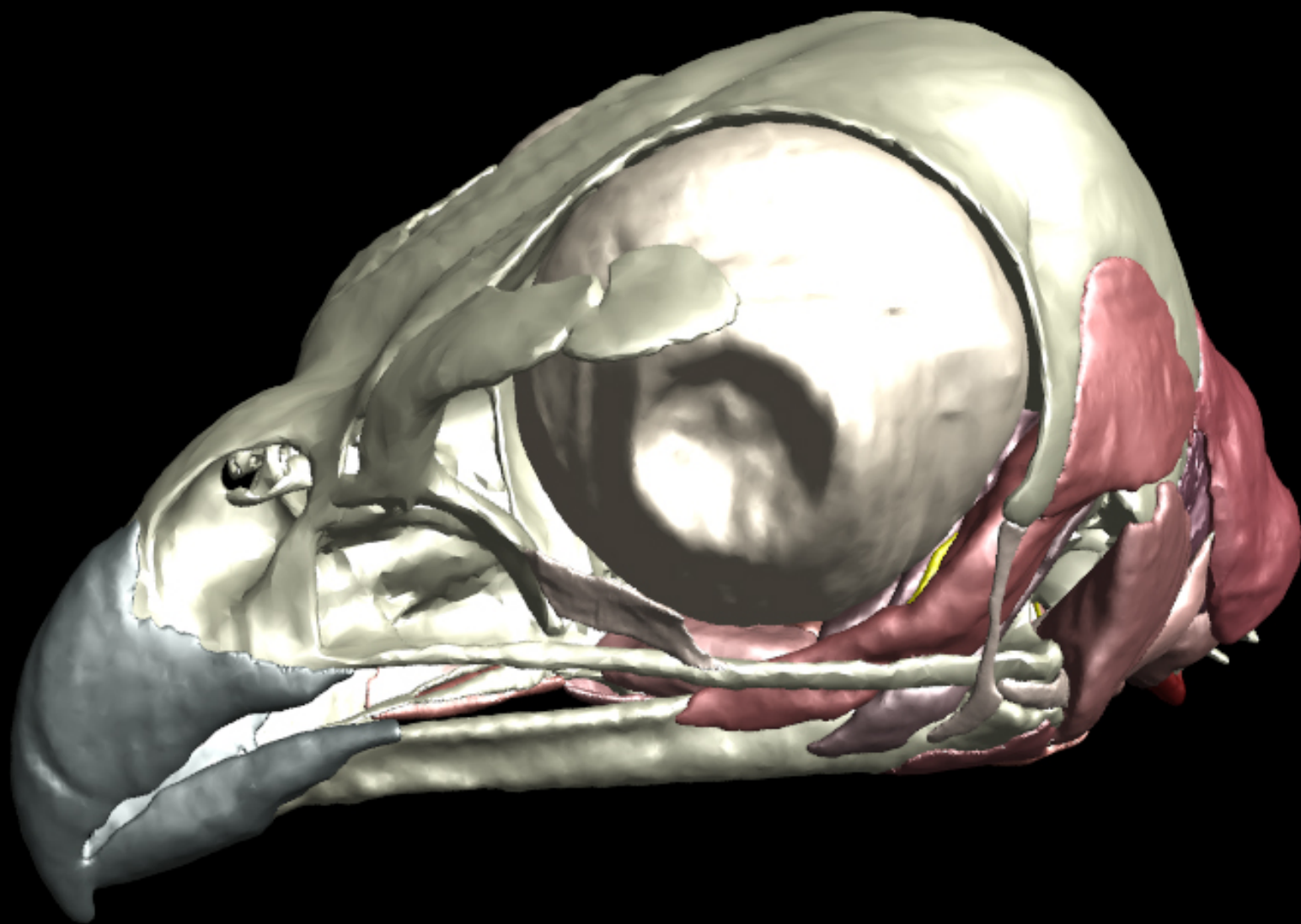

Supplement: Fig S1 — Interactive 3D pdf showing the digitally segmented hardand soft-tissue structures of the Common Buzzard Buteo buteo.[.pdf] [file joa0224-0412-sd1.pdf]
